# The costs of preventing the spread of respiratory infection in family physician offices: a threshold analysis

**DOI:** 10.1186/1472-6963-7-181

**Published:** 2007-11-13

**Authors:** William Hogg, David Gray, Patricia Huston, Wei Zhang

**Affiliations:** 1 Department of Family Medicine, University of Ottawa, Ottawa, Canada; 2 The CT Lamont Primary Health Care Research Center, the Élisabeth Bruyère Research Institute, 43 Bruyère Street, Ottawa, ON K1N 5C8, Canada; 3 Department of Economics, the University of Ottawa, Canada; 4 Surveillance, Emerging Issues, Education and Research Division, Ottawa Public Health, Ottawa, Canada

## Abstract

**Background:**

Influenza poses concerns about epidemic respiratory infection. Interventions designed to prevent the spread of respiratory infection within family physician (FP) offices could potentially have a significant positive influence on the health of Canadians. The main purpose of this paper is to estimate the explicit costs of such an intervention.

**Methods:**

A cost analysis of a respiratory infection control was conducted. The costs were estimated from the perspective of provincial government. In addition, a threshold analysis was conducted to estimate a threshold value of the intervention's effectiveness that could generate potential savings in terms of averted health-care costs by the intervention that exceed the explicit costs. The informational requirements for these implicit costs savings are high, however. Some of these elements, such as the cost of hospitalization in the event of contacting influenza, and the number of patients passing through the physicians' office, were readily available. Other pertinent points of information, such as the proportion of infected people who require hospitalization, could be imported from the existing literature. We take an indirect approach to calculate a threshold value for the most uncertain piece of information, namely the *reduction *in the probability of the infection spreading as a direct result of the intervention, at which the intervention becomes worthwhile.

**Results:**

The 5-week intervention costs amounted to a total of $52,810.71, or $131,094.73 prorated according to the length of the flu season, or $512,729.30 prorated for the entire calendar year. The variable costs that were incurred for this 5-week project amounted to approximately $923.16 per participating medical practice. The (fixed) training costs per practice were equivalent to $73.27 for the 5-week intervention, or $28.14 for 13-week flu season, or $7.05 for an entire one-year period.

**Conclusion:**

Based on our conservative estimates for the direct cost savings, there are indications that the outreach facilitation intervention program would be cost effective if it can achieve a reduction in the probability of infection on the order of 0.83 (0.77, 1.05) percentage points. A facilitation intervention initiative tailored to the environment and needs of the family medical practice and walk-in clinics is of promise for improving respiratory infection control in the physicians' offices.

## Background

There is a paucity of empirical evidence in the literature about actual intervention strategies to improve respiratory infection control practices and analyze the efficiency implications for health policy. Prevention, especially within health care settings, has assumed paramount importance in the fight against respiratory infection. Since influenza is typically transmitted by droplets and contact routes, there are precautions that can be taken to reduce its transmission [1-3]. Interventions designed to prevent the spread of respiratory infection within family physician (FP) offices could potentially have a significant positive influence on the health of Canadians. While there are costs associated with the implementation of any intervention, the benefits stemming from the outcomes of such interventions have the potential to outweigh them.

However, there are few evaluations of outreach facilitation that have studied the net costs of delivering interventions of this nature that exist in the literature. An exception is a study authored by Hogg, Baskerville, and Lemelin [4], which consisted of a randomized, controlled, field trial of an intervention aimed at improving preventative care tailored to the needs of participating family practices. It demonstrated the effectiveness of a multi-faceted outreach facilitation in improving overall preventative care performance. It is the first analysis of cost consequences of an outreach facilitation intervention of which we are aware, and it indicated that the cost savings attributable to the reduction in inappropriate testing on the one hand, and increases in appropriate testing on the other hand, may outweigh all of the intervention costs. Those authors argued that a costly intervention that achieves success may be preferred on a cost-benefit basis to a cheaper one that demonstrates very little or has no lasting effect.

While based on an original and a very different application, this current study employs a similar approach to investigating the resource allocation implications of another type of outreach facilitation intervention that was designed to prevent the spread of respiratory infection within FPs' offices. Evidence from a systematic review has shown that influenza transmission occurs primarily by the droplet and short-distance contact routes [1]. The best practices promoted by the intervention are the droplet and contact precautions, which are described presently. From a clinical perspective, improvement in adoptions of best practices prevents the respiratory virus transmission and therefore, is likely to reduce transmission rates.

## Methods

### Intervention program

Our particular case consists of an outreach facilitation intervention designed to improve respiratory infection control practices in community-based FP offices. It was conducted in the City of Ottawa and delivered by five public health nurses. To our knowledge, it was the first facilitator-based intervention to promote respiratory infection control guidelines. Although the intervention has been documented in detail elsewhere [2,5], we provide a summary of the intervention and its outcomes in this paper.

A total of 53 family medicine practices participated in this pre-post intervention observational study, and all 53 completed the study intervention. Of the 143 participating physicians, 110, or 77 % of them, completed all or part of the pre-intervention questionnaire. The objective was to determine the effectiveness (in terms of compliance) of a short-term intervention to facilitate the incorporation of best practices in respiratory infection control in primary care offices. A mnemonic was developed for both the nurses and physicians to summarize the best practices by the acronym "MASKS" (**M**ask for the patient with cough and a fever, **A**lcohol gel hand sanitization, **S**eating of potentially infectious patient apart from others, "**K**leen"- Disinfection of hard surfaces and **S**ignage).

The intervention commenced with the public health nurse facilitators providing the baseline audit feedback on the respiratory infection control practices in the participating family physicians' practice to physicians and to other practice staff. Physicians were presented directly (and other staff either directly or indirectly through the physicians) with evidence-based best practices and a facilitative "tool kit". This tool kit contained colourful signage outlining best practices for respiratory infection control, signage demonstrating proper hand-washing techniques and use of alcohol-based gel, a reference list of major guidelines sources and web sites, four infection control articles, a box of procedural masks, wall-mounted alcohol gel dispensers with refills, alcohol gel pumps, and hospital-grade disinfectant wipes. During the five-week intervention with their assigned recruited practices, the facilitators worked independently. Throughout the intervention the facilitators corresponded with the project team daily and attended scheduled weekly meetings to share information and strategies.

In order to measure outcomes, four respiratory control activities for an ambulatory office were viewed as the primary indicators of effective respiratory infection control: 1) Signage posted in or about the waiting room; 2) The receptionist giving masks to patients having a cough and/or fever; 3) Instructing patients having a cough and/or fever to use alcohol gel to clean their hands; and 4) Requesting patients having a cough and/or fever to sit at least one meter away from others. Professional nurse auditors were deployed once to obtain data before the intervention and once six weeks after the intervention. The auditor sat for an hour in the waiting room of the physicians' offices and noted the presence or absence of the four respiratory control activities listed just above. They also inquired as to how often potentially contaminated areas were cleaned with disinfectants, and if alcohol-based hand gels were used in examining rooms. The auditors were blinded to the outcome measures and aware only of data gathering requirements. In order to separate the intervention from the data collection, the physicians, office staff and facilitators were blinded from the outcomes and were not informed of the presence of the auditors.

Statistically significant differences between before and after the intervention were observed for all four of the primary outcome measures: 67.3% (95% CI: 54.1%–80.5%), 48.1% (34.0%–62.1%), 54.7% (38.9%–70.5%) and 34.6% (20.1%–49.0%), respectively. Overall, the number of practices that applied all of the four audited primary prevention measures was 3.8% (0%–9.1%) prior to the intervention and 52.8% (38.9%–66.7%) following the intervention (p < .001), demonstrating a 49 (35.1–63.0) percentage point increase in the adoption rate of best practices. This study demonstrated that facilitation of a multi-faceted intervention by public health nurses successfully promoted best practices in respiratory infection control in primary care practices. However, it did not consider health-related outcomes before or after the intervention.

### Cost analysis

We conducted a cost analysis of the respiratory infection control intervention. A standard cost-benefit analysis or cost-effectiveness analysis could not be conducted in this case due to the absence of information on health-related outcomes. As supportive information, we also attempted to evaluate a threshold value for the intervention's effectiveness that could justify the costs incurred by the intervention in terms of the potential cost savings. Standard methodological approaches can be found in Drummond et al. [6] and Muennig [7]. We determined the explicit costs of the intervention from the perspective of the provincial government, which is responsible for financing health care in Ontario. The potential cost savings for this intervention referred to the costs of medical care averted due to the improved respiratory infection control practices that reduce the probability of infection in the physicians' offices. These implicit cost savings can include the cost of health care provider visits by patients experiencing illness symptoms, the cost of medical tests and procedures, and the cost of hospitalizations that were avoided.

### Intervention costs

The actual explicit costs of the intervention over 5 weeks were gathered from the *Public Health Budget Rationale *(2004) for the inputs of labour, auditing services, supplies, facilitator travel, and honoraria that compensated the practices for the time diverted from normal activities. Labour costs referred to the salaries and benefits of the five nurse facilitators and of the 0.5 full-time-equivalent project manager. The audit costs included the costs of the audit itself, involving feedback both before and after the intervention provided to the practices, as well as the traveling costs of the auditor. Supply costs referred to the costs of the tool kits provided by the facilitators for each practice. An honorarium was paid to each FP practice site for the time it spent participating in the project.

In addition to those variable costs, which vary directly with the number of practices that participate, it is important to include the fixed costs of the intervention, which consisted primarily of training the nurse facilitators. The investment in training generated returns extending well beyond the 5-week period of execution of the intervention. The amortization period for recovering the cost of training is much longer than this time frame for the initial intervention, as the skills obtained from training can be utilized again in subsequent years. The initial training cost should therefore be distributed across the estimated useful life of the investment item, taking into account the discount factor. We selected a discount factor of 5 percent as recommended by other papers containing cost analyses [4,6,7]. In addition to the discount rate, we have to select the length of the time period over which to amortize the training cost, which should be related to the life span of the training. As Desai [8] pointed out in his application to obstetrics, often the analyst must assume a length for the useful life span, but this is often initially unclear. Existing research from the field of organizational behaviour indicates that the payoffs stemming from a one-time, up-front investment in employer-paid training for human resources intervention tend to decline after four years [9,10]. Due to the fact that the facilitators received their training over a two-week period, we adopted a somewhat shorter amortization period for the cost of their training by assuming that it is valid for 3 years.

The cost of the training of the five nurse facilitators was amortized over a 3-year life span at a discount rate of 5% based on the training expenses that were initially incurred at the beginning of the intervention. In another scenario, we included the entire training cost into cost analysis instead of amortizing it, which would imply that the training has no value after the current season. Results were also presented at discount rates of 0% and 8% in the mathematical summary which details the discounting process (see Additional file 1).

The other costs listed in the public health budget rationale, such as recruiting participating practices, office assistance, and projection management were not included because these were costs incurred for this particular research pilot project rather than those of the intervention. Those costs would not arise in the facilitation intervention implementation if it were to be adopted on a widespread basis.

All of the direct costs were presented in micro detail for the 5-week period over which the intervention was executed, both in terms of total levels and on the basis of costs per practice. As such, the cost estimates that we generated should generalize to similar projects in other geographic areas that are on either larger or smaller scales. We have made some assumptions regarding how a facilitation program might be organized in order to deal with evaluating the costs of training the facilitators. Our outreach facilitation program is most likely to be effective if delivered during or just before the peak season for respiratory infections (i.e., September, October, and November). Hence our training activities would ideally be applied for 3 months per year over 3 years, generating a cumulative total of 9 months of utilization. While the program would aim to introduce proper respiratory infection control practices to be followed all year round, the medical practitioners might be more interested just prior to the influenza season. Therefore, although the training remains valid for years into the future, we envisaged that the program would be delivered during that 3-month period every year. We nevertheless also produced estimates based on the scenario for which the intervention is executed year-round.

### Cost savings

While the explicit costs of implementing this intervention can be assessed with accuracy, it is much more difficult to estimate the implicit cost savings because of the lack of information regarding a key event, namely the reduction in the probability of spread as a direct result of the intervention. We assume without solid evidence that improved infection control reduces the respiratory infection rate at physicians' offices, but we certainly do not know how by much the probability of infection changed after the intervention. In order to generate an accurate estimate of the total health-care costs averted by this intervention, one would require the following pieces of information: i) the incidence or frequencies of transmission at physicians' office, ii) the effect of the intervention in reducing those rates, iii) the probabilities of the various potential health outcomes that could arise given infection, and iv) the cost of the treatments associated with those outcomes. With the exception of item iv), these pieces of information were not available. Drawing from several data sources in the literature, we therefore adopted an indirect approach to estimate the potential health-care costs that might be averted as a result of the intervention, and we attempted to make a case that the potential benefits were large relative to the explicit intervention costs.

There are a range of treatments for different influenza patients according to the seriousness of the infections. The patient who is infected with influenza may rest at home, visit an emergency room, or be hospitalized. If the patient only needs care at home, he or she may request sick leave from his or her job. In such a case, cost arises from the patient's perspective or the societal perspective (from the lost output) but not from the Ministry of Health's perspective. Another possibility is that a few patients die from influenza, but it is impossible to attach a precise value for the cost of death. Therefore, we only took the intermediate events of outpatient visits and hospitalizations into account in estimating the avoided costs.

The costs denominated in US dollars (as they were presented in some studies that we cited) were converted into Canadian dollars by the current exchange rate [11], and costs from data in prior years were adjusted for inflation and denominated in 2004 constant dollars using appropriate component of the Consumer Price Index [12] where necessary.

### Threshold analysis

The underlying approach for the cost analysis of the intervention involves an efficacy rate, which is defined as the *decrease in the probability of transmission *that is attributable to the intervention. We could not evaluate this quantity, but we could evaluate the threshold value that would render the intervention beneficial, which was judged to be worthwhile if: Cost savings – intervention cost > = 0.

The cost savings attributable to the intervention were expressed as follows: (Cost of hospitalization for a flu patient*number of flu cases avoided due to the intervention in the physician's office*proportion of the infected people who were hospitalized) + (Cost of outpatient visit for a flu patient* number of flu cases avoided due to the intervention in the physician's office*proportion of the infected people who had an outpatient visit). Note that infected individuals who were hospitalized or who had an out patient visit may or may not have passed through the FPs' practices; there are other modes of infection besides transmission in these clinics.

The second element in each of the terms in parentheses, which is a counterfactual, can be expressed as: the number of flu cases avoided in the physician's office due to the intervention = number of patients visiting the physician's office* (probability of contracting influenza in that office without the intervention – probability of contracting influenza in that office with the intervention). Substituting that expression into the primary equation yields the following expression after a slight algebraic manipulation: (probability of contracting influenza in the office without the intervention – probability of contracting influenza in the office with the intervention) = intervention cost/[number of patients visiting the physician's office*(cost of hospitalization*proportion of infected people who were hospitalized + cost of outpatient visit*proportion of infected people with an outpatient visit)].

A critical element for this calculation is transmission rates for influenza in settings such as physicians' offices. While there are articles in the literature dealing with the incidence of transmission of certain viruses within the general population, we were unable to find research pertaining to the incidence of transmission within physician offices or similar locations involving close contact with the public, such as waiting rooms, emergency rooms, and school busses. We searched for papers on Medline, CINAHL and EMBASE by the key words "Influenza or flu, and Transmission or infection, and Bus or waiting room or emergency room or emergency department or physician office", and we also asked for help from several experts in this area to search for the requisite information. We did locate some information regarding the incidence of transmission of influenza during airline flights. In our judgment, however, these figures are not reliable estimates of the rate of infection with and without the intervention that would occur in a FP's office.

In light of that source of uncertainty, our approach was to calculate an estimated value for the left side of the above expression (i.e the reduction in the likelihood of infection) that represents a threshold value for the minimum efficacy of the intervention such that the potential cost savings of the intervention outweigh its costs. We solve that expression for the lowest possible value at which the net costs of the intervention would be negative. If the efficacy of the intervention is any lower than that value, its net costs would be greater than 0.

## Results

### Intervention costs

Table 1 presents the number of hours of intervention work activity and the percentage of total hours spent at the 53 medical practices by the facilitators. The total number of hours worked was 875 (25 days × 5 facilitators × 7 hours/day). In Table 1, it should be noted that the time spent on "other" needs to be removed from the analysis, as that labour time was not allocated to the project. Therefore, the total hours for the five public health nurse facilitators spent on the intervention should be the figures listed under the "Total" label minus those listed under the "Other" label, which worked out to a total of 617 hours. On average, they spent approximately 11 1/2 hours at each practice for which they were responsible. Given a yearly salary of one nurse facilitator of $47,876 (in the Ottawa area) and an annual total of 1,950 hours worked in one year, the hourly wage rate of one nurse facilitator was $24.55. This generated a labour cost per practice of $285.80. In the 5-week intervention period, the labour costs (for time actually worked) for all five nurse facilitators combined amounted to $15,147.35 ($24.55 × 617 hrs).

**Table 1:** Number of hours of intervention work activity and the distribution of total hours*

**Activity **	**PF 1 # (%)**	**PF 2 # (%)**	**PF 3 # (%)**	**PF 4 # (%)**	**PF 5 # (%)**	**Total # (%)**
**Implementing site services**						
Audit & ongoing feedback	3 (0.3)	23 (2.6)	2 (0.2)	5 (0.6)	3 (0.3)	36 (4.1)
Planning & consensus building	5 (0.6)	10 (1.1)	11 (1.3)	6 (0.7)	12 (1.4)	44 (5.0)
Waiting time	3 (0.3)	4 (0.5)	4 (0.5)	3 (0.3)	3 (0.3)	17 (1.9)
**Travel and administration**						
Travel	19 9 (2.2)	24 (2.7)	23 (2.6)	22 (2.5)	29 (3.3)	117 (13.4)
Administrative duties relating to PH/FM	57 (6.5)	55 (6.3)	82 (9.4)	119 (13.6)	91 (10.4)	403 (46.1)
Other: vacation, sick time	89 (10.2)	59 (6.7)	53 (6.1)	21 (2.4)	37 (4.2)	258 (29.5)
						
Total	175 (20)	175 (20)	175 (20)	175 (20)	175 (20)	875 (100)

* **For 53 practices by the 5 outreach facilitators**

**Notes: **Total hours = 875 (25 days × 5 facilitators × 7 hours/day). **PF refers to Practice Facilitator**. All figures have been rounded to the nearest hour. The total for each row (in the right-most column) indicates the number of raw hours worked by all five facilitators for each activity. The figure listed beside it in parentheses indicates the share of the 875 hours that is accounted for by that activity. The total for each column (in the bottom row) indicates the number of raw hours worked by each facilitator on all activities combined. The figure listed beside it in parentheses indicates the share of the 875 hours that is accounted for by each facilitator.

The total costs for the intervention are presented in Table 2. The third column provides the data on the costs of the outreach facilitator intervention denominated in 2004 dollars on the basis of the 5-week period during which they actually worked. The fourth column contains similar data, except that all of the costs were estimated on the basis of a 13-week (or 3-month) time period that corresponds to the peak flu season. These figures were calculated based on the assumption that the 5 facilitators would work at the same pace for 13 weeks instead of 5 weeks, and would thus visit approximately 138 practices. Similarly, the figures in the fourth column of Table 2 were based on the assumption that the 5 facilitators would work at the same pace for an entire year, and would thus visit approximately 551 practices. The difference between these three scenarios consists of a pro-rating of all of the variable costs while holding the training costs fixed. The figures in the last column were exactly same as those in the third column except for the training cost. The training cost presented in the last column was not amortized over 3 years, which accounts for the approximately threefold increase in the training costs coupled with no change in the other costs. The intervention costs amounted to a total of $52,810.71 that was actually incurred over the 5-week intervention, $131,094.73 per flu season, or $512,729.30 per calendar year.

**Table 2:** Total costs for the intervention

**Cost item**	**Rationale**	**Amounts (2004) (Five weeks)**^1^	**Amounts (2004) (3 months)**^2^	**Amounts (2004) (per year)**^3^	**Amounts (2004) (Five weeks)**^4^
Salaries and benefits	Five Nurse Facilitators	$15,147.35	$39,383.11	$157,532.44	$15,147.35
	0.5 full-time equivalent project manager	$4,831.54	$12,562.00	$50,248.02	$4,831.54
Total training	Course, experts, test practices	$3,883.20*	$3,883.20*	$3,883.20*	$10,562.30
Audit	Auditors and travel	$11,100.00	$28,860.00	$115,440.00	$11,100.00
Supplies	Tool kits	$7,472.18	$19,427.67	$77,710.67	$7,472.18
Facilitator travel		$2,426.44	$6,308.74	$25,234.98	$2,426.44
Honorarium	Each practice site was compensated for time lost	$7,950.00	$20,670.00	$82,680.00	$7,950.00
**Intervention costs**		**$52,810.71**	**$131,094.73**	**$512,729.30**	**$59,489.81**

*The training cost was amortized over 3 years using 5% discount rate, and therefore the training cost for each calendar year was $3,883.20, as shown in the additional file 1. This training cost for five facilitators would be totally fixed for a 3-year period. Even if these 5 facilitators were to conduct this activity for the entire flu season, and thus serve more than 53 practices, this cost would not change.

^1 ^Costs for the 5-week intervention based on the actual 5-week length of the project.

^2^Assume that intervention lasts 3 months (13 weeks) during flu season. All figures except the training cost were obtained by converting the 5-week totals (that apply to our particular intervention) listed in third column to weekly rates and then multiplying by 13.

^3^Assume that intervention lasts one year (52 weeks). All figures except the training cost were obtained by converting the 5-week totals (that apply to our particular intervention) listed in third column to weekly rates and then multiplying by 52.

^4 ^Costs for the 5-week intervention based on the actual 5-week length of the project and the training cost was not amortized.

In order to extrapolate these cost figures to other geographical areas, the distinction between the variable costs and the fixed costs plays an important role. The variable costs that were incurred for this 5-week project amount to $48,927.51, which is the sum of all of the costs listed in the third column of Table 2 with the exception of the training costs. This corresponds to approximately $923.16 per participating practice. In order to estimate the corresponding variable cost for a similar project elsewhere, one can extrapolate that estimate by multiplying by the number of practices involved. This estimate is premised on salaries in effect in the Ottawa area, as well as travel distances for a fairly large urban area.

The (fixed) training costs must be calculated in a different fashion, however. As explained in the mathematical summary (see Additional file 1), we calculated an annual value of $3,883.20 for the training costs. This figure is equivalent to $73.27 for each participating practice. Had these five facilitators worked for the entire 13-week flu season, the total training costs would still be $3,883.20, but many more practices could have been involved, thus lowering the per-practice training cost to $28.14 ($3,883.20/138 practices). If these same facilitators were to be assigned to this project on a year-round basis, the per-practice training costs would become one quarter of the prior figure, or $7.05, because the nurses work 4 times longer during the year.

### Cost savings

The first element that we obtained for the expression for averted costs was the number of patients that passed through the offices of the participating physicians, and were therefore at risk of becoming infected. In pre-intervention questionnaire, physicians were asked how many patients they typically see per half-day, from which we may estimate the number of patients visiting the physician offices during a 5-week period. 103 physicians responded to the question, and the mean value was 14.55. Imputing this value to all of the physicians that were covered in our intervention, approximately 104,033 patients visited the 143 participating physicians over 5 weeks.

The figures for the costs of treating influenza patients were drawn from several sources. Hogg, Baskerville and Lemelin [4] performed a cost savings analysis associated with administering influenza vaccine in the elderly. They obtained the estimated cost of an emergency room visit due to influenza from Jacobs and Hall, which was approximately CN $76.00 in 1999 or CN $84.00 in 2004 [4,13]. This cost was virtually identical to the costs for an outpatient visit reported in other studies [14,15]. Thus, for the value of the out-patient component of health care that includes visits to the physician's office and to the emergency room, we used the figure of $84.00. The Ontario Case Costing project provided the total cost of hospitalization for the following treatments: pneumonia (CN $4,462 in 1999 or CN $4,931.89 in 2004), chronic respiratory conditions (CN $4,445 in 1999 or CN $4,913.10 in 2004), and congestive heart failure (CN $5,417 in 1999 or CN $5,987.46 in 2004) for patients 65 years of age and older [4].

We turn next to the proportion of people infected with influenza that ended up being hospitalized. An estimate of this proportion can be obtained by dividing the hospitalization rate among all subjects with influenza (regardless of where it was contracted) by the proportion of all subjects who become infected (regardless of where it was contracted). The latter quantity can be thought of as the illness or transmission rate of the influenza. In an analogous fashion, an estimate of this proportion of infected people who had an out-patient visit can be obtained by dividing the out-patient rate among all subjects with influenza by the proportion of all subjects who become infected.

Unfortunately, we could find no paper in the literature that provided values for the hospitalization rate or the outpatient visit rate given that a patient has influenza. In order to obtain rough estimates of these quantities, we borrowed heavily from the paper by Nichol [16] that dealt with vaccination against influenza. By a systematic literature review, the author obtained estimates of 'the hospitalization rate due to influenza and its complications', 'outpatient visit rate due to influenza and its complications', and 'the influenza (and its complications) illness rate' among healthy working adults aged between 18 and 64 years. Nichol also derived from the Monte Carlo simulation the *difference *of the hospitalization rate (as well as the outpatient visit rate and the illness rate) for influenza and its complications between unvaccinated and vaccinated subjects. However, the influenza's complications were widely defined in Nichol's paper. In our analyses, we focused on only influenza and pneumonia associated with influenza. Therefore, by assuming that vaccination is 100% effective in preventing episodes of influenza (and pneumonia associated with influenza), we used the number of vaccinated individuals as a proxy for the number of non-infected subjects. In this respect, the three difference rates reported by Nichol can be interpreted as each of these three incidence rates due to influenza only (and pneumonia associated only with influenza). Therefore, we used these differences, 0.026% (95% probability interval (PI): 0.011%, 0.043%), 2.5% (1.2%, 4.5%), and 5.5% (3.2%, 9.0%), as our estimates for 'the hospitalization rate due to influenza only', 'outpatient visit rate due to influenza only', and 'the influenza illness rate', respectively.

When we inserted these values into the expression for the proportion of infected patients who ended up hospitalized, we obtained a value of 0.00472 (PI: 0.00344, 0.00478), and the value for the proportion of infected patients who had an outpatient visit was 0.4545 (PI: 0.375, 0.5). Inserting all of the figures that we obtained above back to the primary expression for the cost savings, and combining that information with the value for the explicit costs of intervention, the efficacy of the intervention (probability of contracting influenza in the office without the intervention – probability of contracting influenza in the office with the intervention) was equal to 0.83% (PI: 0.77%, 1.05%). The implication is that the threshold value for the efficacy at which the cost savings of the intervention barely outweigh the costs was 0.83%. The goal would thus be to reduce the probability of infection occurring in FPs' offices by at least 0.83%. In addition, if we included the non-amortized training cost into analysis, the threshold value rose slightly to 0.93%. The figures that entered into the calculations are presented in Table 3.

**Table 3:** Threshold calculations

**Variable**	**Value**	Value (sensitivity analysis)*
Cost per hospitalization	CN $4,931.89	CN $4,931.89
Cost per outpatient visit	CN $84	CN $84
Probability of infected people who were hospitalized	0.472% (0.344%, 0.478%)	0.472% (0.344%, 0.478%)
Probability of infected people who had any outpatient visit	45.45% (37.5%, 50%)	45.45% (37.5%, 50%)
Number of patients visiting physician offices in 5 weeks	104,033	104,033
Total intervention costs for 5 weeks	CN $52,810.71	CN $59,489.81
**Efficacy of Intervention**	**0.83% (0.77%, 1.05%)**	**0.93% (0.87%, 1.18%)**

*Total intervention costs included the training cost without amortization.

## Discussion

This paper has provided detailed information on the costs of an outreach facilitation initiative designed to prevent the spread of infectious diseases by promoting best practices in respiratory infection control in primary care practices. We have generated accurate estimates of the explicit costs of implementing such a program on a per-practice basis, which permits the extrapolation of these unit costs to other geographical domains. We have also provided some preliminary estimates of the potential cost savings to the health-care system. Due to the lack of knowledge about the frequency of respiratory infection occurring at physicians' offices, particularly an estimate of the reduction in the probability of infection attributable to the intervention, we did not have enough evidence to evaluate precisely the benefits of the intervention. As an alternative approach, we undertook a threshold analysis to estimate a threshold value of the efficacy that could render the intervention cost saving. Based on our conservative estimates referring to direct savings in the form of health-care costs averted, there are indications that the outreach facilitation intervention program would result in cost savings if it could achieve a reduction in the probability of infection at the physician offices on the order of 0.83 percentage points. This implies that if we assume that there was a 1.00% chance of contracting influenza in FP offices without intervention, to achieve the efficacy rate of 0.83%, the probability of contracting influenza in FP offices with intervention would be 0.17%, representing a large relative risk reduction in influenza transmission in FP offices. On the other hand, if we assume a higher probability of contracting influenza in FP offices without intervention, such as 5%, to achieve the targeted efficacy rate of 0.83%, the probability of contracting influenza would be approximately 4.2%, representing a smaller relative risk reduction in influenza transmission in FP offices.

Moreover, in addition to the direct cost savings to the health care system that may be realized, there are potential indirect cost savings associated with our intervention as well, such as the potential to avoid disastrous human loss and suffering caused by viruses such as the Severe Acute Respiratory Syndrome (SARS). The scope of the influences of the infectious diseases such as SARS and influenza extend far beyond the costs that were mentioned above, especially in the health-care and tourism sectors. The total costs in terms of lost production of the SARS epidemic to Toronto's economy had been estimated to be $1 billion, and the estimate for the economic cost for all of Canada was around $1.5 billion [17]. Within the health care sector, the indirect costs borne by non-SARS patients were enormous. SARS affected all health-care workers – especially those on the front line – and delayed "non-emergency surgeries" such as organ transplants and cancer radiation [18]. According to Ontario Health Minister Tony Clement, as of June 27, 2003, SARS had cost Ontario's health-care system $945 million, which was spent mostly on special supplies and added health-care workers needed to protect health-care workers, as well as on constructing specialized SARS clinics and isolation rooms [17]. This in turn had a huge impact on non-SARS related health care system utilization, both due to diversion of resources as well as severe stress amongst the health care providers. For instance, a study comparing the periods before and during the SARS outbreak in the GTA and non-GTA areas by Woodward et al. found the greatest impact of SARS on reduction in the utilization of inpatient and outpatient hospitalization, diagnostic testing, physician and emergency department visits, use of prescription drugs, intensive care bed availability, and cardiac care during April 2003 to May 2003 [19]. Avoiding such negative consequences implies that our intervention may also generate implicit or indirect cost savings.

## Conclusion

The 5-week intervention costs amounted to a total of $52,810.71. The results of the cost analysis suggest that the intervention can be cost saving because the 0.83% point reduction of the probability of influenza at the physicians' offices appears to be a feasible target for the effectiveness of the studied intervention. A facilitation intervention tailored to the environment and needs of the family practice and walk-in clinics is of great promise for improving respiratory infection control in the physicians' offices. Future research to conduct further economic evaluations of such an intervention based on adequate data – particularly in relation to infection incidence rates and the ability to lower them – would aid in important public health policies and administrative decision-making on implementing preventive care guidelines.

## Competing interests

The author(s) declare that they have no competing interests.

## Authors' contributions

William Hogg and Patricia Huston conceived the intervention, provided the data, participated in the study design and in critical revisions of the manuscript, and contributed to all other aspects of the study. David Gray contributed to the cost evaluation study design, assisted with the calculations, and thoroughly revised the manuscript. Wei Zhang acquired the economic data, performed the cost evaluation analysis and interpretation, and drafted the first pass of the manuscript. All authors read and approved the final manuscript.

## Pre-publication history

The pre-publication history for this paper can be accessed here:



## Supplementary Material

Additional file 1 Mathematical summary – amortization of training costs. Click here for file
