# Supplementary material for: The costs of preventing the spread of respiratory infection in family physician offices: a threshold analysis
Source: BMC Health Serv Res. 2007 Nov 13;7:181. doi: 10.1186/1472-6963-7-181 (PMC2204002; doi:10.1186/1472-6963-7-181)
Supplement: Additional file 1 — Mathematical summary – amortization of training costs. [file 1472-6963-7-181-S1.doc]

# Additional file 1: Mathematical summary

**Amortization of training costs**

1. The total up-front costs of the training of the five nurse facilitators were amortized over a 3-year life at an interest rate of 5 % in order to generate an estimate for the costs over one year that take account of the fact that much of the up-front training costs can be allocated to later periods.

**Training cost (present value) = One year cost/(1+r) + One year cost/(1+r)^2^ + One year cost/(1+r)^3^**

**⇒ One year cost = Training cost *r/(1-(1+r)^-3^)**

**⇒**

**One year cost = Training cost *0.05/(1-(1+0.05)^-3^) = $10562.30*0.05/0.136 = $3,883.20**

2. The same exercise is carried out using a higher interest rate of 8 %, which generates a higher figure

**One year cost = Training cost *0.08/(1-(1+0.08)^-3^) = $4,098.53**

3. The same exercise is carried out using a lower interest rate of 0 %, which generates a lower figure. In this instance, exactly one-third of the cost is allocated over each of the 3 years.

**One year cost = Training cost / 3 = $3,520.77**

4. The other costs associated with the intervention include salaries and benefits, audit, supplies, facilitator travel and honorarium are adjusted from 5 weeks to 3 months and one year.

**3 months = 13 weeks**

**3 month costs = 5-week costs x 13 / 5**

**One year costs = 5-week costs x 52 / 5**
